# A Bibliometric Analysis of Refugee Health Publications in the Nursing Field by Visual Mapping Method

**DOI:** 10.1192/j.eurpsy.2024.1279

**Published:** 2024-08-27

**Authors:** G. Ozturk, G. Dikeç, A. K. H. Seren

**Affiliations:** Nursing, Fenerbahce University, Istanbul, Türkiye

## Abstract

**Introduction:**

Millions of people worldwide are forced to migrate to another country and nurses are the key professional for providing necessary health care to this population. Providing nursing care to refugees or immigrants requires diverse transcultural professional competencies based on standardized guidelines.

**Objectives:**

This study was aimed to examine the bibliographic characteristics of quantitative studies conducted on refugees in the nursing field.

**Methods:**

The data were obtained from articles scanned in the Web of Science Core Collection database. The 1672 articles that were published between 1980-and 2023 and met the inclusion criteria were analyzed using VOSviewer and Microsoft 365 Excel software. The PRISMA 2020 Checklist was used for reporting.

**Results:**

Most publications were made in 2020. The United Kingdom, the United States, Canada, and Australia have the highest number of publications, citations, and international cooperation. Additionally, “mental health” is one of the most used keywords in the studies.

**Conclusions:**

The findings show the importance of empowering nurses working in this field, especially in determining the needs related to mental health services for refugees. The increased migration rates and the growing need for refugee health care highlighted the importance of investment in nursing research within this field. Nurses and researchers should aim to establish partnerships and share best practices with the leading countries. Furthermore, nurses require specialized training to competently evaluate and provide nursing care and mental health services to this vulnerable population. Policymakers must prioritize international collaboration, equitable healthcare, and the integration of mental health services within healthcare systems to improve refugee health and reduce barriers between them and health services.

**Disclosure of Interest:**

None Declared

